# Supplementary material for: A single-cell atlas to map sex-specific gene-expression changes in blood upon neurodegeneration
Source: Nat Commun. 2025 Feb 25;16:1965. doi: 10.1038/s41467-025-56833-7 (PMC11862118; doi:10.1038/s41467-025-56833-7)
Supplement: Supplementary file 1 — Supplementary Information [file 41467_2025_56833_MOESM1_ESM.pdf]

A single-cell atlas to map sex-specific gene-expression changes in blood upon neurodegeneration

Supplementary Information

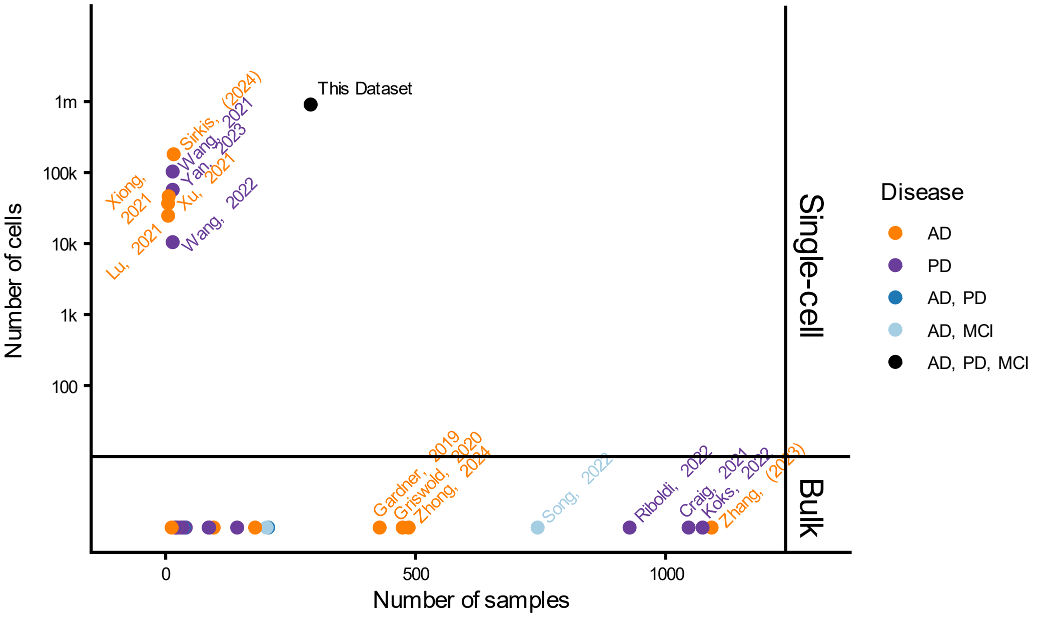

Supplementary Fig. 1: Previously published datasets of PBMCs in Alzheimer’s and Parkinson's disease.
Number of cells and samples of the datasets, labeled with the name of the first author and year of publication. For pre-prints the years were put in brackets. References to the datasets are available in Supplementary Table 2.

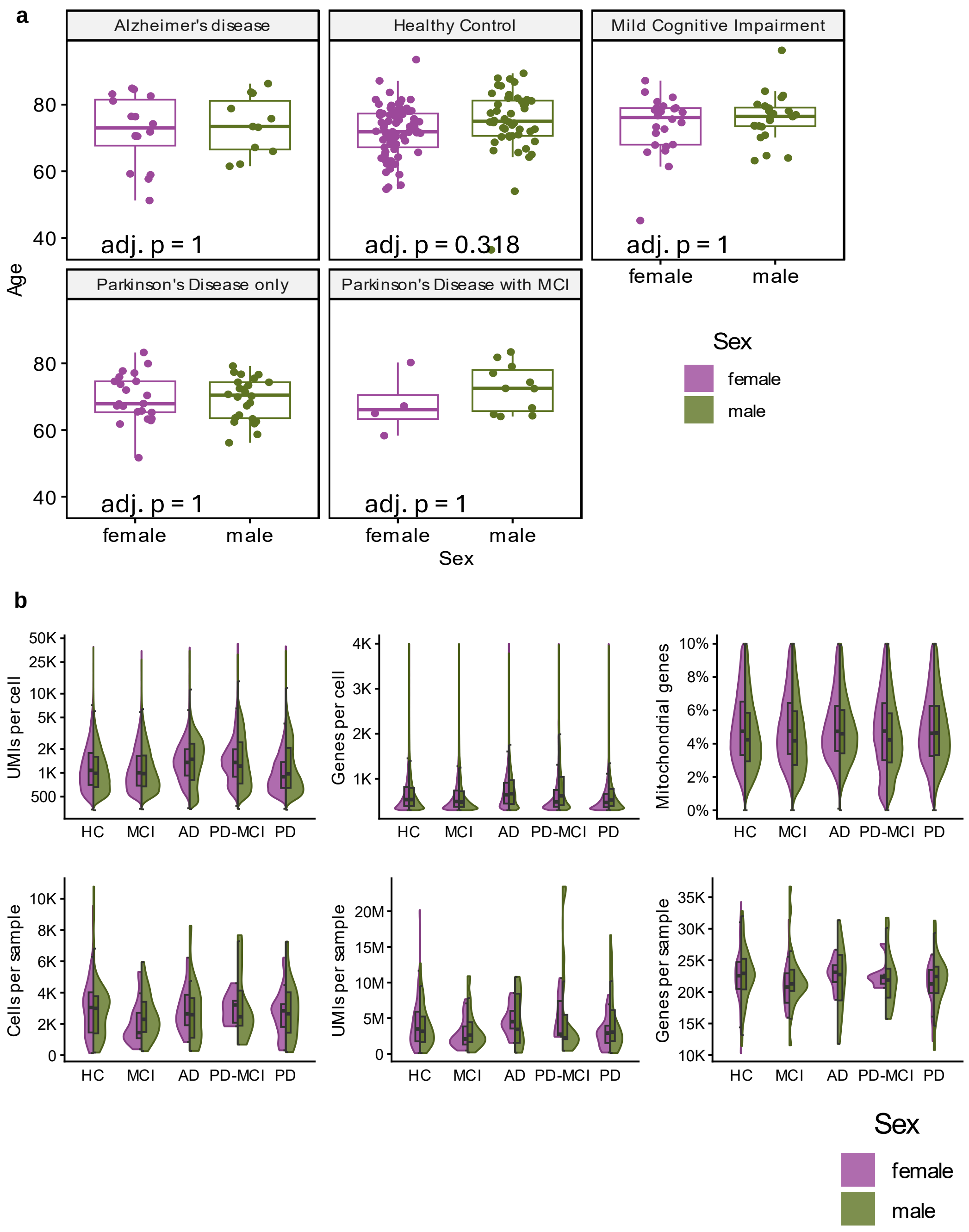

Supplementary Fig. 2: Distribution of Quality-Control Measurements and meta-data per patient group and sex.
a, Distribution of age per disease and sex shows no significant difference between the age of male and female patients in the different diseases (Sample size see Table 1). Boxes are defined as in Figure 1b.
b, Distribution of the quality-control measurements per patient group and sex. Violin plots and boxes are defined as in Figure 1b.

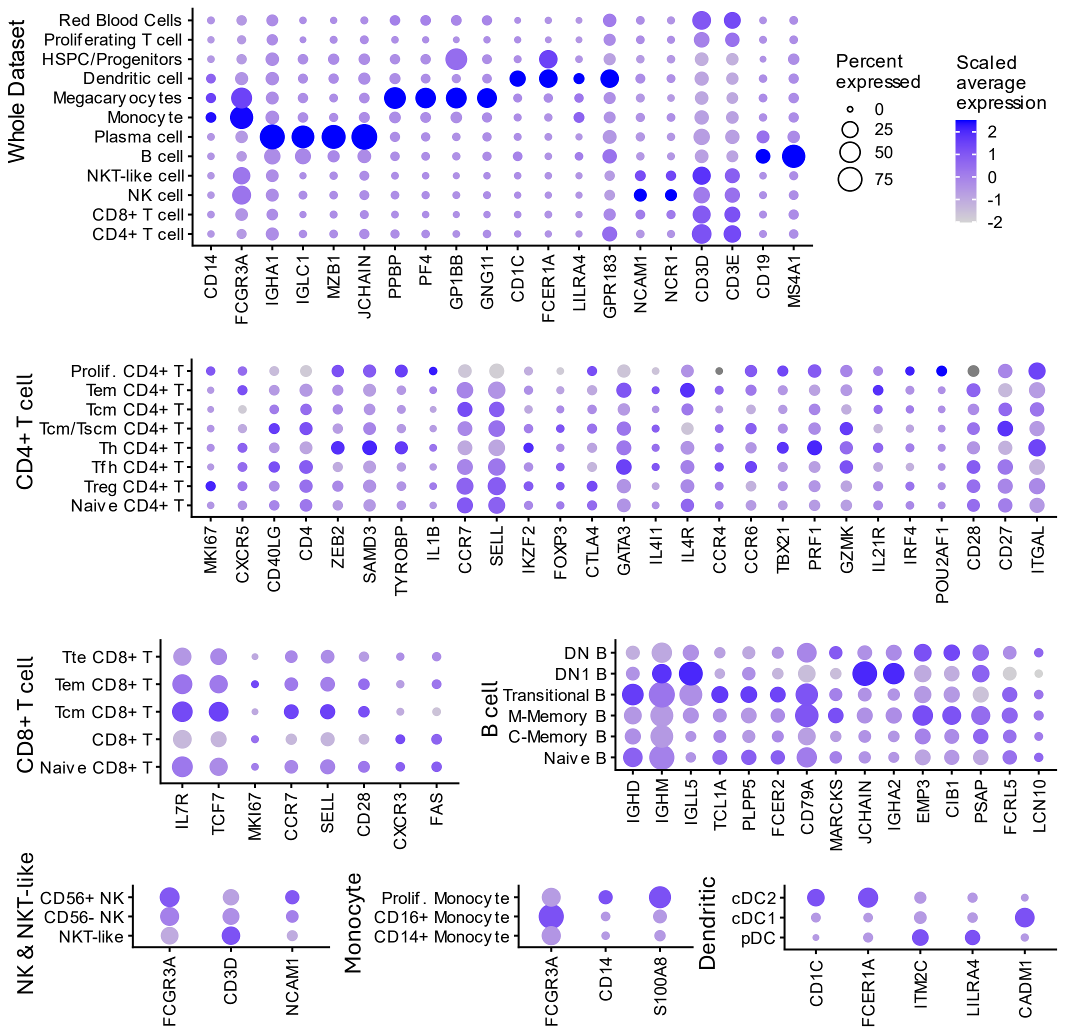

Supplementary Fig. 3: Expression of cell type markers used to classify cells into the 12 general cell types and the markers used to distinguish the sub-groups.

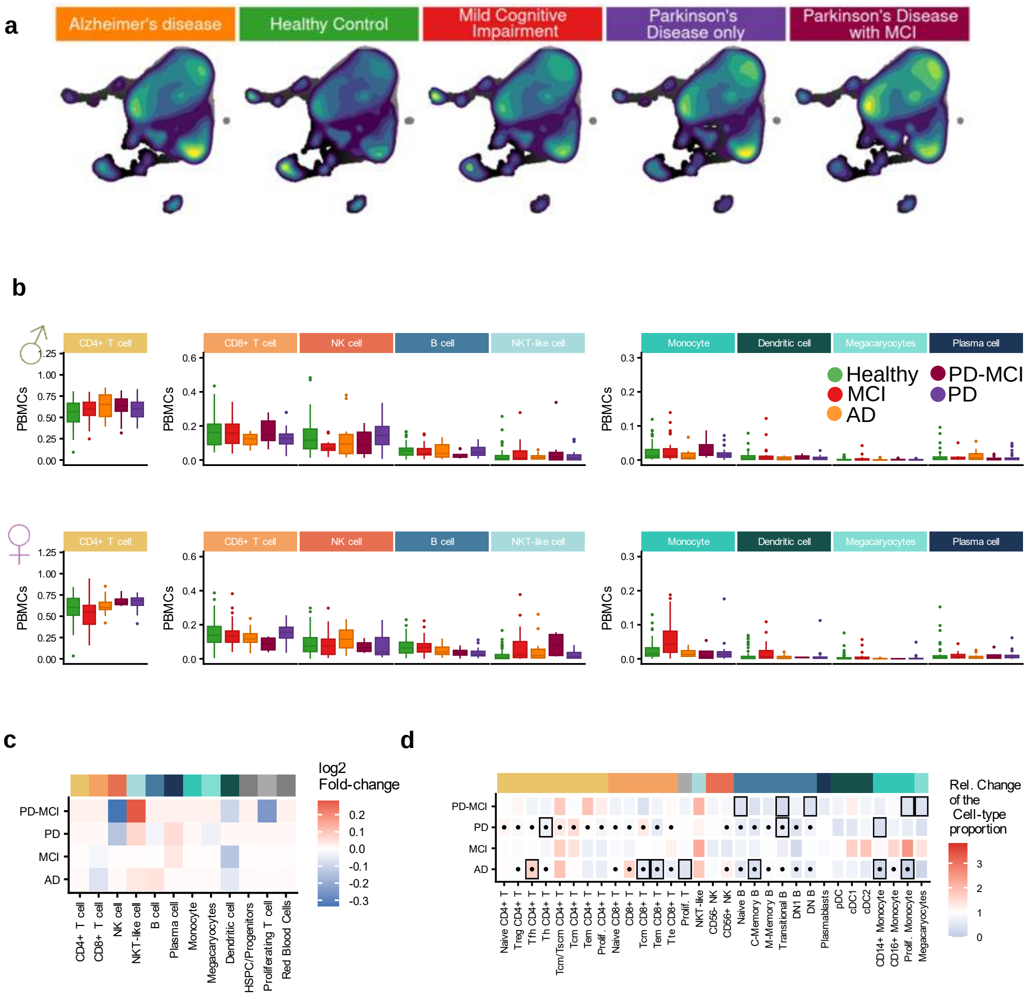

Supplementary Fig. 4: Changes in the cell type composition

a, Density UMAP embedding for the different disease group. Each embedding shows the spots with the highest density in yellow and with the lowest density in blue.

b, Disease-specific changes in the cell type proportions per sample and patient group for males and females. Boxes are defined as in Figure 1b.

c, Fold changes of the cell type composition changes determined using scCODA using both male and female samples.

d, Relative change in the cell type-proportion in % between Healthy and the diagnosis groups for the different cell types reveals sex-specific changes. Significant values are marked with a frame around the tile, previously described changes from the literature are marked with a dot. (n_{AD}= 27, n_{PD}= 46, n_{MCI}= 48, n_{PD-MCI}= 15, n_{HC}= 121)

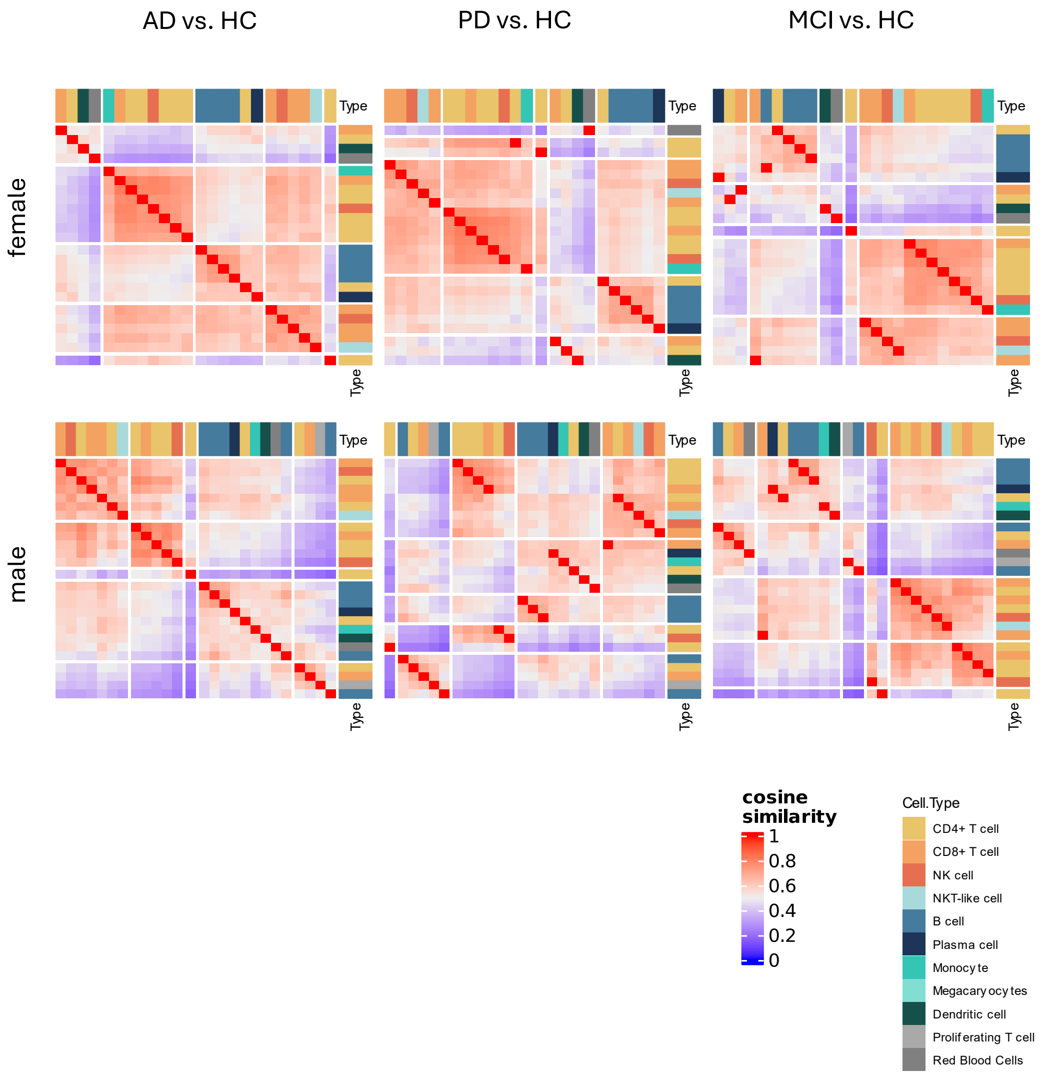

Supplementary Fig. 5: Similar patterns of deregulation across different cell types.
Cosine similarity between the gene-lists ordered by fold-change show similar patterns of de-regulation within the different T cell clusters and within the different B cell clusters.

a

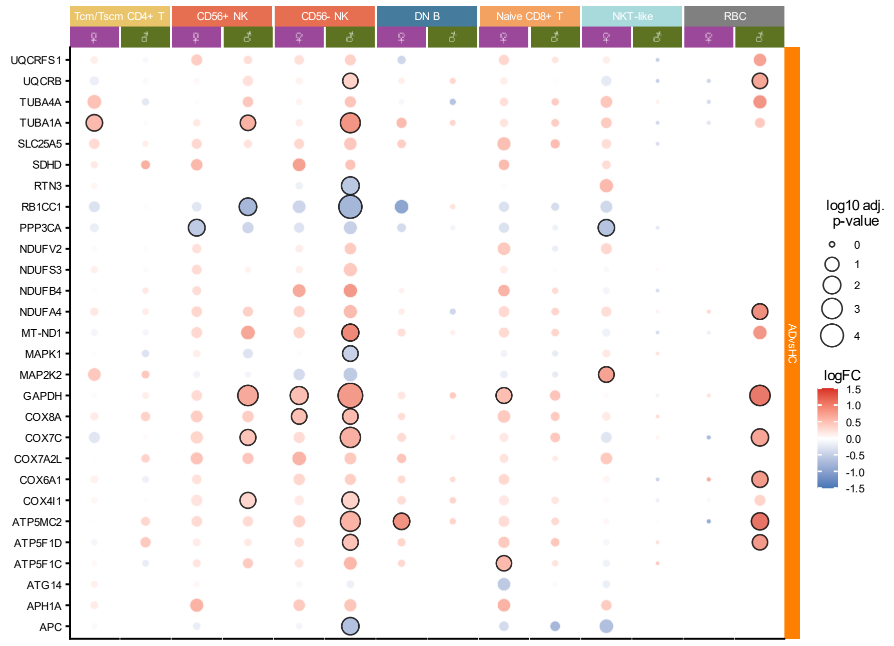

b

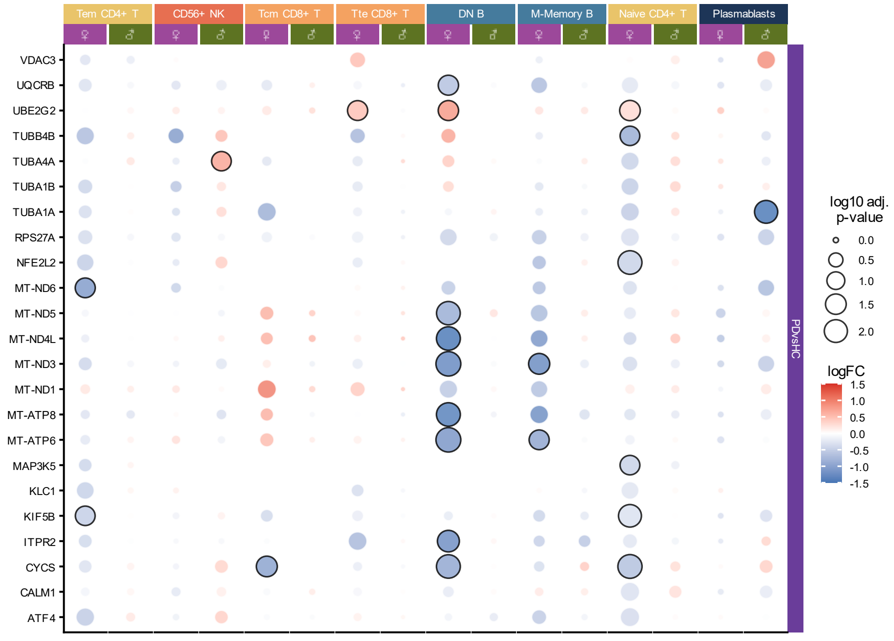

Supplementary Fig. 6: Gene-expression of genes from the KEGG Alzheimer's and Parkinson's pathways.

a, Changes in gene-expression for the most frequently significantly de-regulated genes of the KEGG Alzheimer-pathway are similar in the different cell types and sexes. Significant values are marked by a black circle. **b,** For the genes of the KEGG Parkinson's pathway, the changes in gene-expression show more differences between the different cell types and sexes.

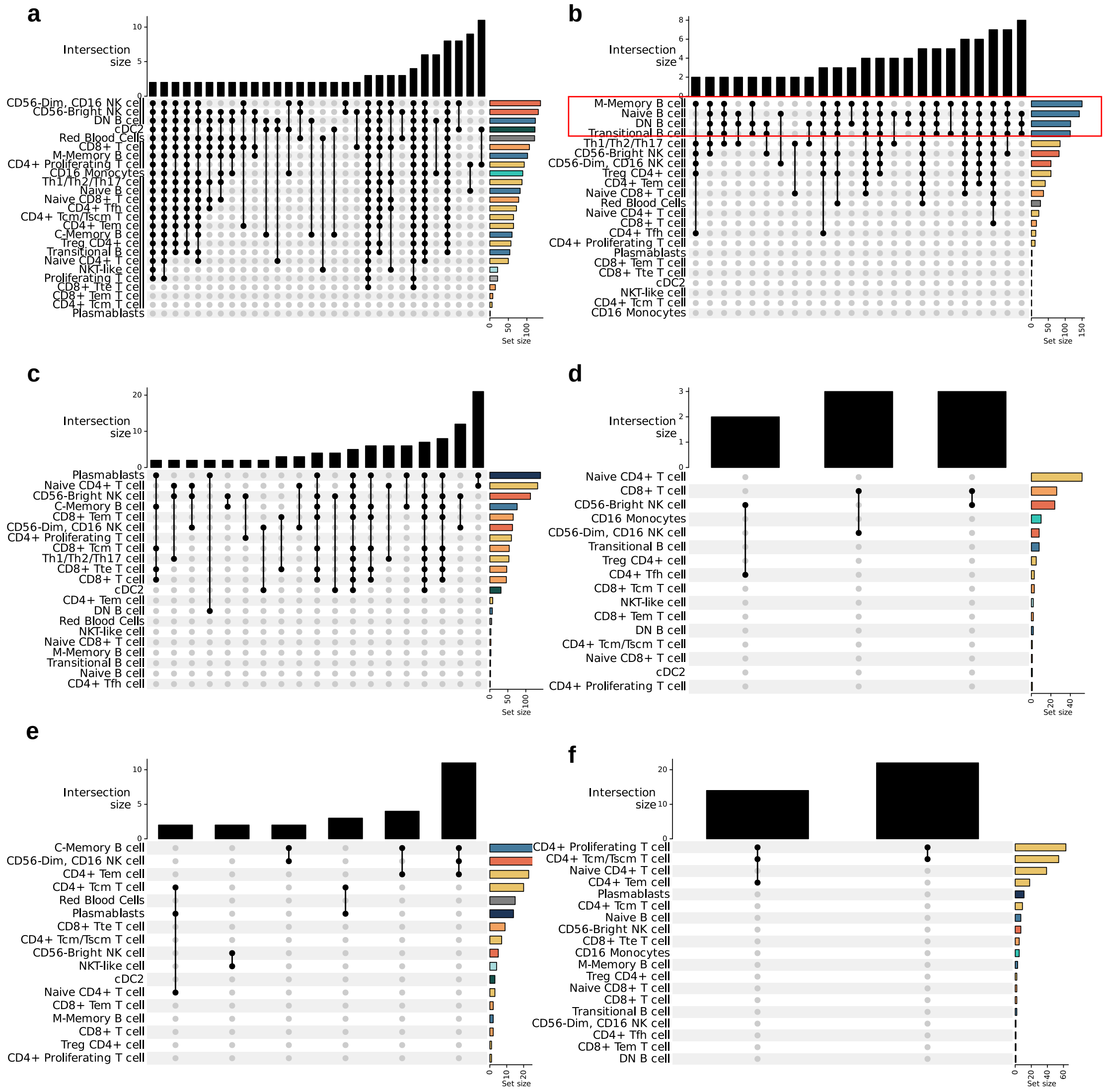

Supplementary Fig. 7: Overlap of the enriched and depleted pathways un the different cell-types.

a-f, Upset Plot showing the overlap of the enriched and depleted pathways in the different cell-types in AD (a) and (b), in PD (c) and (d) and in MCI (e) and (f) in male patients (a, c, e) and female patients (b, d, f).

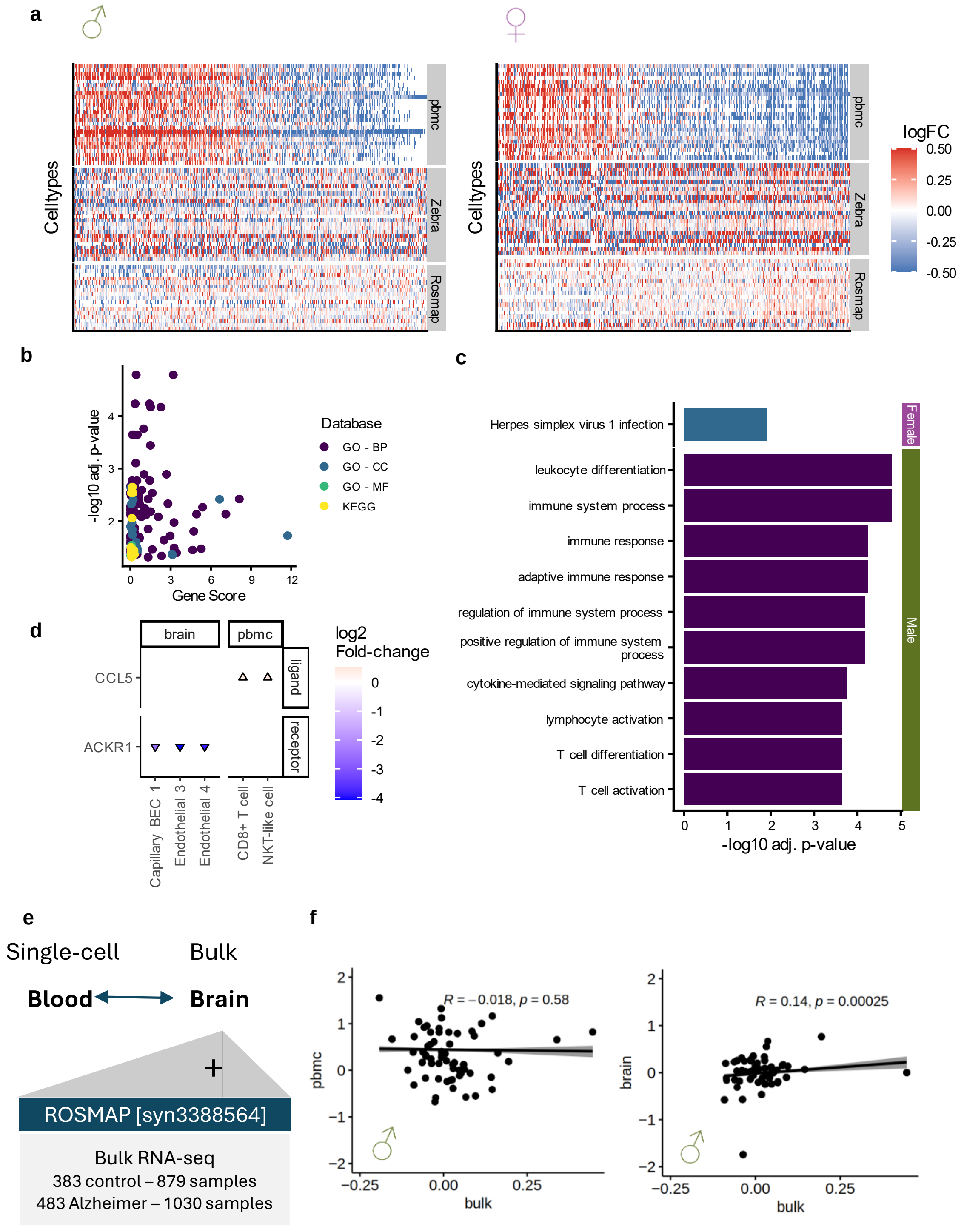

Supplementary Fig. 8: Comparison of the changes in PBMCs and Brain. **a,** Fold-changes of all significant de-regulated genes between HC and AD in the brain (cortex/prefrontal-cortex) and PBMCs in comparison for both sexes. **b,** Result of the pathway analysis of the de-regulated genes in PBMCs and the brain cells in males and females. **c**, Top 10 enriched pathways with the smallest p-values in males are related to the immune system and in females to Herpes simplex virus 1 infection. **d**, Significantly de-regulation of genes that are part of the CCL signaling pathway in females. **e&f,** Bulk RNA-sequencing dataset (b) used to compare changes found in single-cell data of the brain and blood with changes found in bulk data of male patients (c) (Pearson's correlation, pbmc/bulk: n=933, bulk/brain: n=653).

a

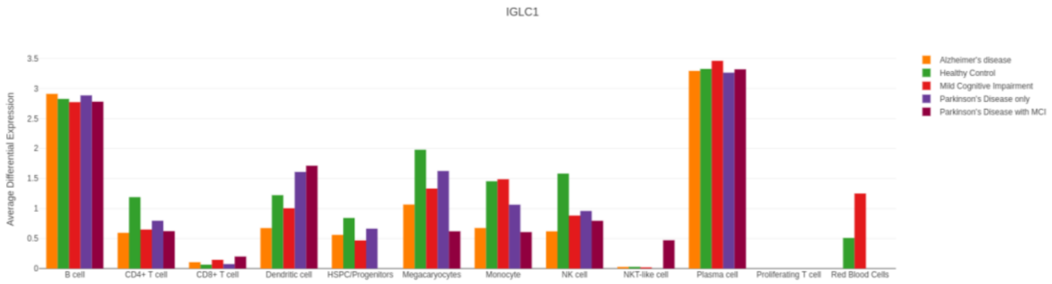

b

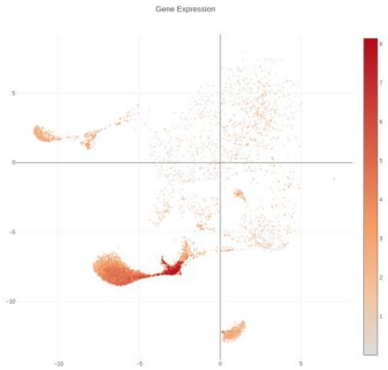

c

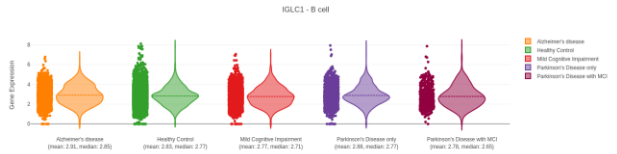

Gene	Cluster Id	Contrast	logFC	p adj.loc	Sex
IGLC1	B cell	ADvsHC	0.811	0.459	female
IGLC1	B cell	PDvsHC	0.613	0.551	female
IGLC1	B cell	MCIvsHC	0.323	0.822	female
IGLC1	B cell	PDMCIvsHC	0.524	0.887	female
IGLC1	B cell	ADvsHC	1.01	0.564	male
IGLC1	B cell	MCIvsHC	0.309	0.826	male
IGLC1	B cell	PDvsHC	0.347	0.889	male
IGLC1	B cell	PDMCIvsHC	-0.242	0.998	male
Gene	Cluster Id	Contrast	logFC	p adj.loc	Sex

Supplementary Fig. 9: Easy data-access and comprehensible visualization in a webserver.

a&b, Screenshots of the visualization of the gene-expression in the UMAP-embedding (a) and as a bar-plot (b) in the webserver. **c,** Violin-plot and table showing the result of the DEG analysis, filtered by cell type (B cells) and gene (IGLC1).

Gene Expression

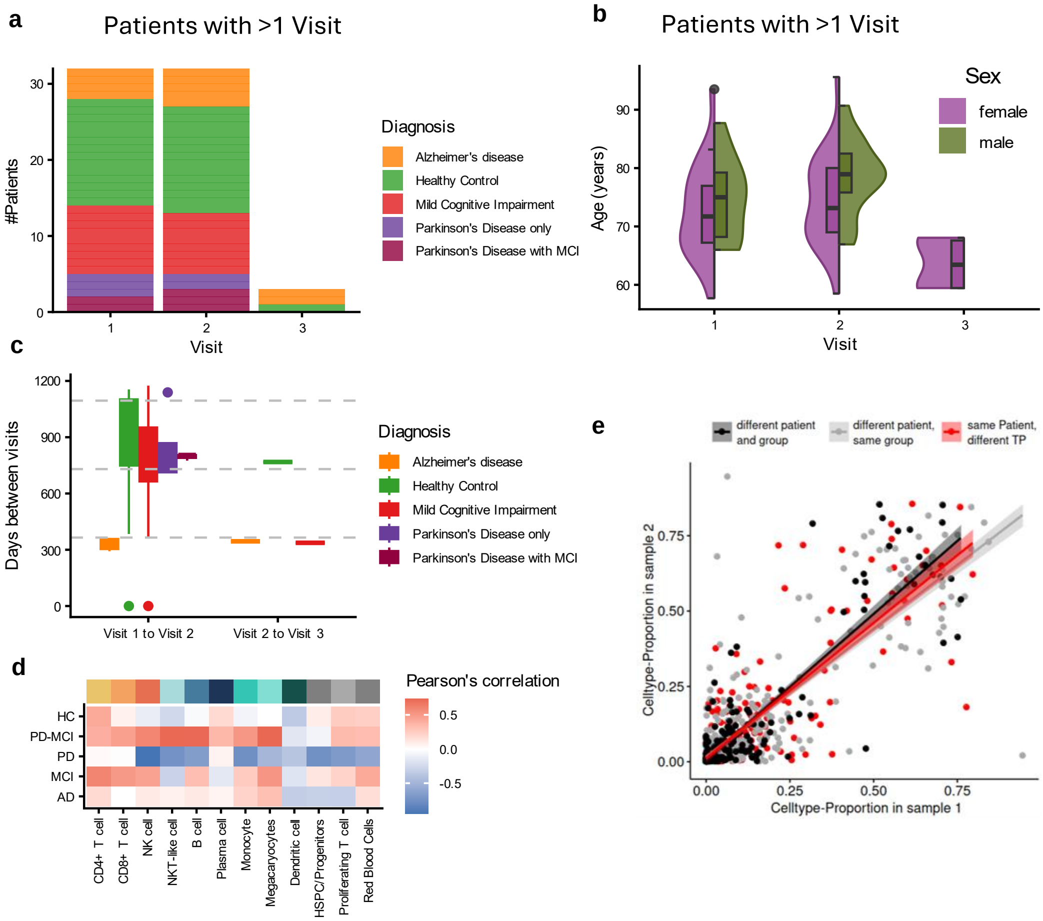

Supplementary Fig. 10: Comparison of gene-expression and cell-type composition across timepoints

a, Number of patients per disease-group at the first, second and third visit. Note that the diagnosis between visits did change in some cases. **b,** Average age of the male and female patients with more than one visit at their first, second and third visit. Violin plots are defined as in Figure 1b. **c,** Number of days between the visits for patients with more than one visit. One, two and three years are marked by a dashed horizontal line. Boxes are defined as in Figure 1b. **d,** Correlation of cell type proportions with the number of the visit. None of the comparisons were significant after adjustment. (number of samples: see (a)) **e,** Comparison of the cell type proportions between samples from the same patients but different time-points and between samples from different patients belonging to either the same or different diagnosis groups (AD/PD and HC) shows that the variance in the cell type composition in a patient is similar to the variance between patients independent of the group they belong to.

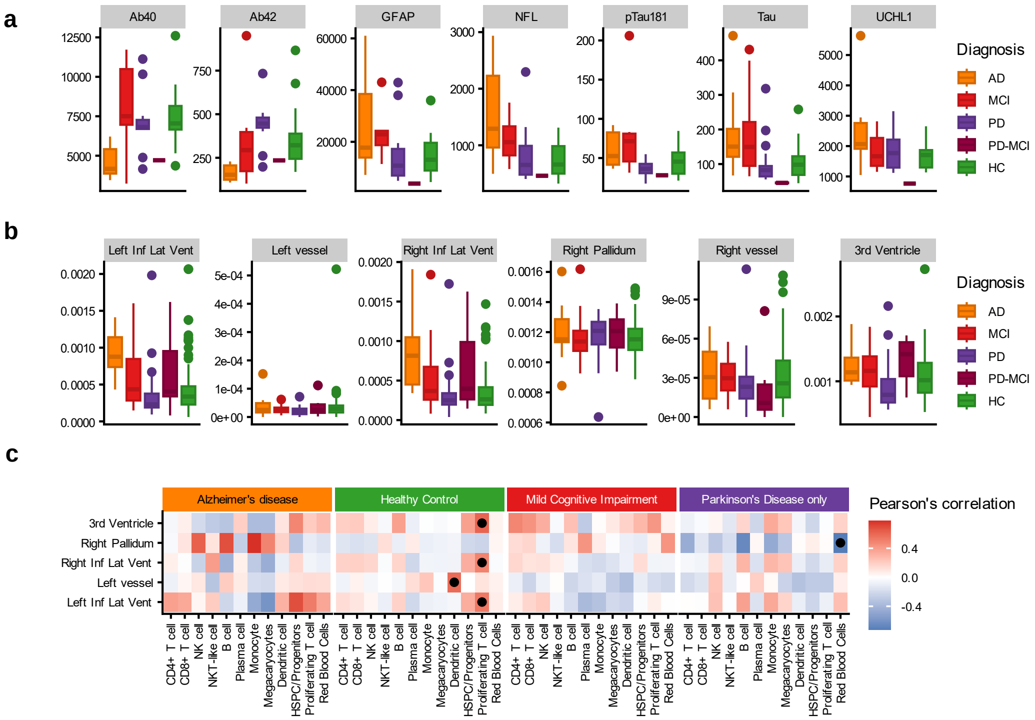

Supplementary Fig. 11: CSF and Brain data in the dataset.

a&b, Level of known AD biomarkers in the CSF (a) and brain volume measurements (b) in patients of the different disease groups. **c,** Correlation between the CSF and brain data and the cell type proportions in the single-cell data. Significant correlations are marked with a dot (n_{AD}= 13, n_{PD}= 29, n_{MCI}= 29, n_{HC}= 88).

Supplementary Table 1: Sex-specificity of Alzheimer's and Parkinson's studies found during the Literature Research

Paper	Tissue (Detailled)	Tissue	Disease	Sex-specific?
Xiong et al., 2021 [1]	Blood/PBMC	Blood	AD	No
Hu et al., 1995 [2]	Peripheral blood lymphocytes (PBL)	Blood	AD and other dementia	No
Pirttilä et al., 1992 [3]	Peripheral blood	Blood	AD	No
Gate et al., 2020 [4]	PBMC	Blood	AD	No
Shad et al., 2013 [5]	Blood	Blood	AD	No
Wang et al., 2019 [6]	Blood	Blood	AD	No
Amin et al., 2020 [7]	PBMC	Blood	AD and Lewy Body Dementia	No
Qian et al., 2022 [8]	Peripheral blood	Blood	AD & MCI	No
Li et al., 2022 [9]	Peripheral blood	Blood	PD	No
Chen et al., 2021 [10]	Peripheral blood lymphocytes (PBL)	Blood	PD	No
Rocha et al., 2018 [11]	Peripheral blood	Blood	PD	No
Garfias et al., 2019 [12]	Peripheral blood lymphocytes (PBL)	Blood	AD & PD	No
Kustrimovic et al., 2018 [13]	Peripheral blood	Blood	PD	No
Sochocka et al., 2022 [14]	Peripheral blood lymphocytes (PBL)	Blood	AD	Yes
Ji et al., 2022 [15]	Peripheral blood	Blood	AD	Yes
Coales et al., 2022 [16]	Microglia, monocyte, macrophages	Blood and Brain	AD	Yes
Patel et al., 2020 [17]	Brain	Brain	AD	No
Felsky et al. 2022 [18]	Brain	Brain	AD	No
Zhao et al., 2015 [19]	Parietal lobe tissues	Brain	AD	No
Moradifard et al., 2018 [20]	Brain	Brain	AD	No
Carlisle et al., 2021 [21]	Peripheral blood monocytes	Blood	PD	Yes
Sommer et al., 2018 [22]	Blood	Blood	PD	No
Mogi et al., 2000 [23]	Substantia nigra	Brain	PD	No
Arif et al. 2010 [24]			PD	No
Lang et al., 2019 [25]	Human iPSC-Based Model of Parkinson's Disease	Model	PD-related phenotypes	No
Varghese et al., 2009 [26]	Blood	Blood	PD	No
Holmes et al., 2016 [27]	Rat Dopaminergic Neuronal Cell Line	Model		No
Maki et al., 2002 [28]	Cerebrospinal fluid (CSF)	CSF	AD	No
Tsugu et al., 1998 [29]	Cerebrospinal fluid (CSF)	CSF	AD	No
Nilsson et al., 2021 [30]	Hippocampus	Brain	AD	No
Kish et al., 1998 [31]	Brain	Brain	AD	No
Butterfield et al. 2010 [32]	Brain	Brain	AD	No
Tsai et al., 2020 [33]	Blood	Blood	AD	No
Dang et al., 2022 [34]	Astrocytes	Brain	AD	No

Supplementary Table 2: Single-cell and bulk RNA-sequencing datasets with Alzheimer's disease (AD), Parkinson's disease (PD) and Mild Cognitive Impairment (MCI)

Paper	Year	Single-cell/bulk	#Samples	#Cells	Disease
Sirkis et al. [35]	(2024)	Single-cell	16	182000	AD
Wang et al. [36]	2022	Single-cell	14	10466	PD
Wang et al. [37]	2021	Single-cell	14	103365	PD
Yan et al. [38]	2023	Single-cell	14	57126	PD
Xiong et al. [1]	2021	Single-cell	6	46244	AD
Xu et al. [39]	2021	Single-cell	5	36849	AD
Lu et al. [40]	2021	Single-cell	5	24679	AD
Zhang et al. [41]	(2023)	Bulk	1092	0	AD
Koks et al. [42]	2022	Bulk	1074	0	PD
Craig et al. [43]	2021	Bulk	1046	0	PD
Riboldi et al. [44]	2022	Bulk	928	0	PD
Song et al. [45]	2022	Bulk	744	0	AD, MCI
Zhong et al. [46]	2024	Bulk	486	0	AD
Griswold et al. [47]	2020	Bulk	474	0	AD
Gardner et al. [48]	2019	Bulk	428	0	AD
Hooshmand et al. [49]	2022	Bulk	205	0	AD, PD
Song et al. [50]	2022	Bulk	202	0	AD, MCI
Panitch et al. [51]	2022	Bulk	179	0	AD
Hemmings et al. [52]	2022	Bulk	143	0	PD
Dressman et al. [53]	2022	Bulk	96	0	AD
Infante et al. [54]	2015	Bulk	87	0	PD
Dhanwani et al. [55]	2022	Bulk	86	0	PD
Infante et al. [56]	2016	Bulk	40		PD
Annesley et al. [57]	2022	Bulk	40		PD
Garofalo et al. [58]	2020	Bulk	36		AD, PD
Carlisle et al. [21]	2021	Bulk	34		PD
Henderson et al. [59]	2021	Bulk	30		PD
Kurvits et al. [60]	2021	Bulk	24		PD
Hu et al. [61]	2020	Bulk	21		PD
Cardona et al. [62]	2021	Bulk	20		AD
Schlachetzki et al. [63]	2018	Bulk	20		PD
Wang et al. [64]	2022	Bulk	12		AD

Supplementary Table 3: Literature reporting genes that were found in Alzheimer’s brain and blood

Gene	Category	Reference
TRG-AS1	AD-general	[65]
ZNF212	Not reported	-
GON4L	Not reported	-
IQGAP2	BBB and Immune	[66]
CYTOR	Not reported	-
CXCR4	AD-general	[67]
CD74	Immune response	[68, 69]
CD37	Plaques	[70]
FOSB	Cognition	[71]
RBM3	Not reported	-
PMAIP1	Mitochondrium	[72]
ITGB2-AS1	Inflammation	[73]
CYTH4	Not reported	-
LILRB1	Microglia	[74]
IFI16	Immune response	[75]
HLA-DQB1	AD-general	[76]
RHOH	Microglia	[77]
ARL4C	Not reported	-
HBB, HBA1, HBA2	AD-general	[78]
SLAMF7	Not reported	-
SCML4	Not reported	-
VIM	Astrocytes	[79]
ANXA1	Inflammation	[80]
MCUB	Mitochondrium	[81]
JUNB	Not reported	-
FAU	Not reported	-
RPL18	Ribosome	[82]
LYN	AD-general	[83, 84]
RRBP1	AD-general	[84]
FTH1, SAT1	Astrocytic Ferroptosis	[34]
ATP5MC3	AD-general	KEGG Alzheimer’s pathway
FYB1	AD-general	[85]
NBPF14	Not reported	-
LGALS1	Microglia/Immune	[86]
CTSW	Immune response	[87, 88]
CD7	Immune response	[89]

Supplementary Note 1: Machine learning of potential biomarker panels using Random Forests

Finding a minimal set of genes that can be used to distinguish healthy from diseased cells allows the easy and fast detection of biomarkers in single-cell data. The state-of-the-art method for biomarker detection in single-cell data is differential expression analysis, which usually outputs a large number of genes making it difficult to find reliable biomarkers. Most methods also assume a linear relationship between gene expression and the experimental condition or covariate and thus consider each gene independently. Our method uses Random Forests and a backwards feature elimination. Random forests are non-parametric models that can handle complex, non-linear relationships between features and the outcome. This allows the identification of gene sets whose expression patterns are characteristics of the disease status of cells.

Feature selection using Random Forests and backwards feature elimination

Feature selection through Random Forests with backwards feature elimination enables identification of compact sets of genes that reliably distinguish between healthy and diseased cells. Starting with normalized gene expression data, an initial Random Forest model is trained using the clinical diagnosis as the response variable. The importance of each gene is evaluated based on its contribution to the model's predictive performance. Genes with the lowest importance are iteratively removed, and the process is repeated on reduced gene sets until only a small number of genes remain. At each iteration, model performance is assessed to determine the subset of genes that best predicts the clinical diagnosis. This approach leverages Random Forests' ability to model complex, non-linear relationships and potentially enables the discovery of robust biomarker panels that characterize disease status while reducing redundancy in gene sets.

Classification of female Alzheimer's patients

All female Alzheimer's patients (16 samples) and all female control patients (73 samples) were used for training and testing the Random Forest based feature selection.

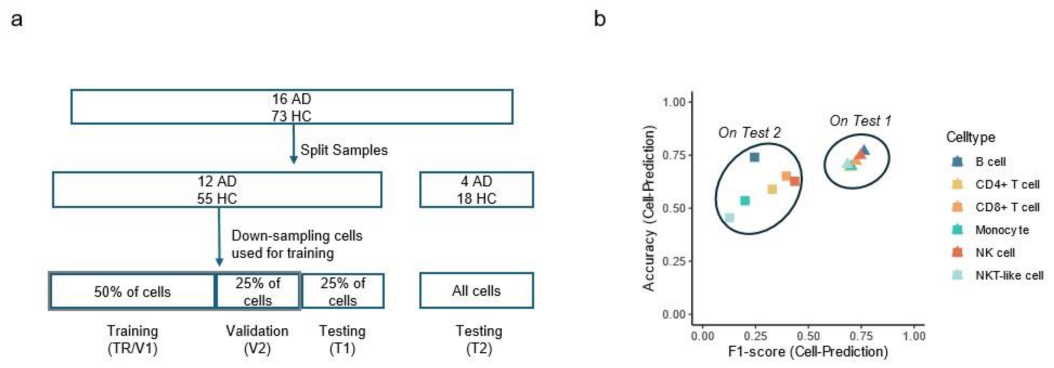

Figure 1: Random-Forest Classification of the Diagnosis using single-cell data a) Initial splitting of the dataset into four different groups. Donors from T2 are independent from the rest, donors from TR/V1, V2 and T1 were not independent. b) Comparison between F1 score and Accuracy of the classification of cells on the Test set T2.

4 Alzheimer's and 18 control patients were kept separate as an independent test set (T2). The cells from the remaining patients were distributed into a Training (TR/V1) a Validation (V2) and a Test set (T1) (see Figure 1a). The cell-level predictions were tested on the Test 1 set and Cell- and Patient-level Predictions on the Test 2 set. Patient-level predictions were not evaluated on Test 1, as the set of patients is not independent from the patients included in the Training- and Validation-data.

Using default parameters to train the Random Forests (ntree = 500, no maximum depth; input: top 2000 highly variable genes) the classification resulted in an accuracy of up to 0.76 and an F1 score up to 0.76 on the Test 1 set (Fig. 1b). On the Test 2 dataset, the accuracy was replaced by the F1 score to account for the unbalanced test set (T2). This resulted in a much lower F1 score in all cell-types on Test2 (mean = 0.28) than on Test1 (mean = 0.71). This indicates that the model might over-train the patients included in the training data.

Hyperparameter-Optimization of the Random Forests

To see if the parameters of the random forest model have an influence on the generalizability of the classification, a grid-based hyperparameter-optimization was performed for the number of trees, the maximal depth and the input feature (gene) set. The number of trees were tested for 250, 500 and 1000. The Maximum depth was selected from 10, 20 and no maximum.

To see if this choice of input features has an influence on the classification and feature selection, different input feature sets were compared. Performing single-cell data analysis on the top highly variable genes (HVGs) is a common approach [90]. Therefore, the top 2000, 4000, 6000 and 8000 highly variable genes were tested as input. The three random feature sets all containing 2000 randomly selected genes were tested and used as a baseline comparison. The last set contained all genes that are expressed in at least 1% of cells in each condition in the training set (LowExpressionFilter) and provided the largest feature set.

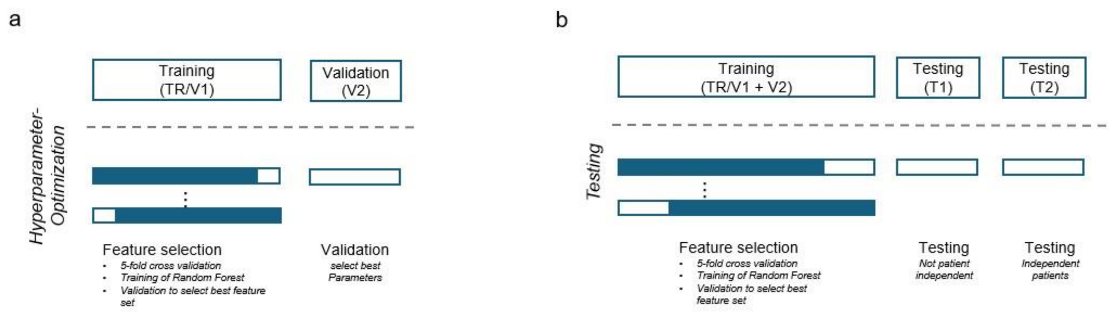

Figure 2: Sub-sets used for Hyperparameter-optimization and testing of the Random-Forest based Feature-Selection. a) Hyperparameter-optimization is performed using TR/V1 and V2. Using different parameters, the feature-selection is performed TR/V1 and evaluated on V2. The best performing hyperparameters are then used to train the final model. b) The feature-selection is performed using TR/V1 and V2 combined. The model is then tested using T1 and T2.

The model was trained on TR/V1 and evaluated on V2 to select the optimal parameters (Fig. 2a). Once the best hyperparameters were selected, TR/V1 and V2 were combined into one bigger Training set (Fig. 2b).

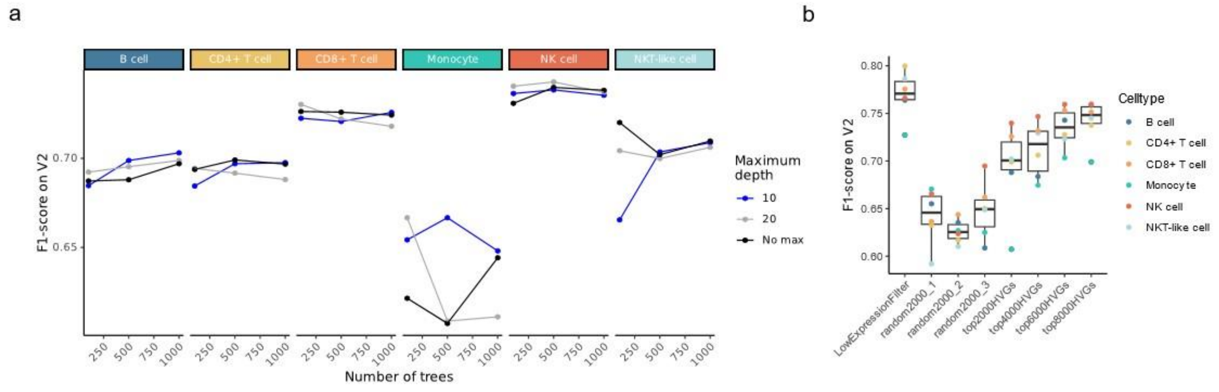

Figure 3: F1 score of AD status predictions on validation set V2 using different hyperparameter (a) and input feature set (b) configurations for feature selection and final random forest models for the cell-level data set split.

The maximum depth and the number of trees showed only a small influence on the F1 score (difference in F1 < 0.03 for CD4+ CD8+ T, B and NK cells). Only Monocytes and NKT-like cells showed a larger variation of the F1 score with different parameters being optimal (Fig. 3a). The use of the low expression filter and the different sets of HVGs yielded gene selections with higher classification performances than the use of random feature sets (Fig. 3b). The F1 score increased with the size of the chosen input gene set, with the use of the low expression filter resulting in the highest mean F1-score on the V2 set across the six cell types.

The overall best combination of parameters is to use all genes expressed in at least 1% of cells in each condition, 500 trees and a maximal depth of 20.

Comparison with different feature selection methods

The Random Forest model was then re-trained with optimal parameters and using TR/V1+V2 for the feature selection.

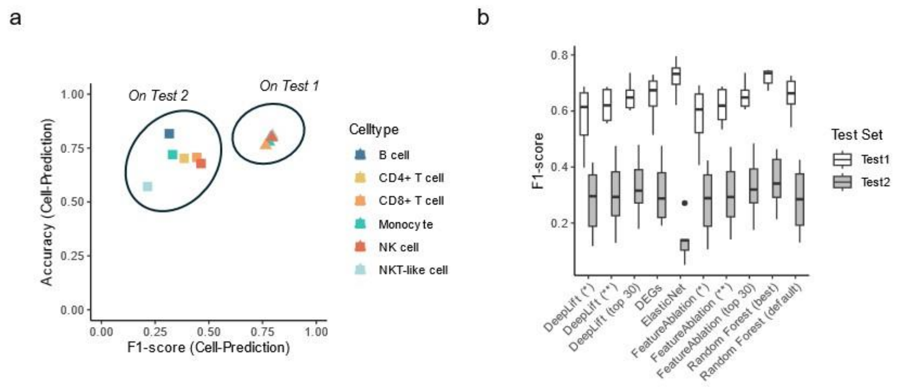

Figure 4: Performance on of the optimized Random Forest-model a) Accuracy and F1 score on Test 1 and Test 2. b) Result of the kNN classification using the feature sets selected by different methods as input. (*): using the same number of top-ranking genes as selected by the random forest approach with default configuration, (**): using the same number of top-ranking genes as selected by the random forest approach with best configuration]

The model achieved an average F1 score of 0.78 in Test 1 and 0.35 in Test 2 (Fig. 4a). Using optimal instead of the default parameters thus did not improve the generalizability of the model.

To see if other machine-learning based feature-selection methods show a better generalizability, the same task was performed using three independent methods: one logistic regression-based method (ElasticNet) and two deep learning-based approaches (DeepLIFT + MLP and FeatureAblation + MLP). The result of the differential-expression analysis (methods see paper) was used as an extra comparison. The classification performance based on the selected feature sets was then evaluated using an independent classification method. The used model is a k-nearest neighbors (kNN) classifier, a simple classifier that evaluates the quality of the selected features independent of the feature selection method.

For all methods the F1 scores for the Test2 set were lower than on Test 1 (Fig. 4b). The best performance was achieved by the Random Forest-based method on CD8+ T cells showing the best performance on Test 2 with an F1 score of 0.42.

Importance of a strict patient split in model training

To ensure that the training is not driven by donor-specific effects, a new dataset split was applied. The dataset was now split by donors and a group k-fold cross validation was used for the training to ensure that cells in training and validation-set originate from different patients (same was applied to ElasticNet).

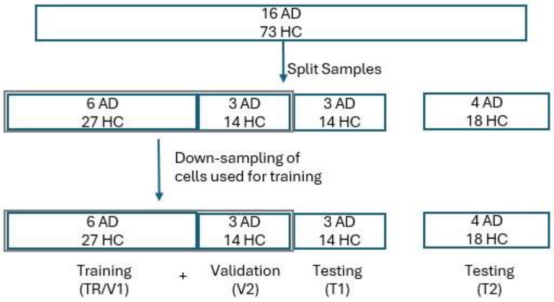

Figure 5: Patient level splitting of the dataset. The dataset was split so the patients do not overlap in training/validation, Test1 and Test2.

The test-set (T2) still contains the same patients and cells as in the previous tests (Fig. 5a). The remaining patients were distributed into TR/V1, V2 and T1 set. The hyperparameter optimization was repeated using the new data split.

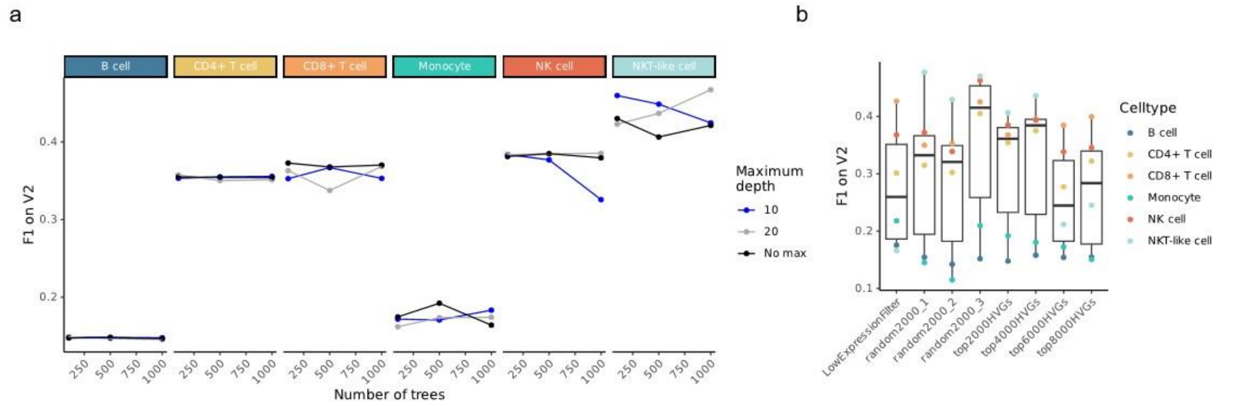

Figure 6: F1 score of AD status predictions on validation set V2 using different hyperparameters (a) and input feature sets (b) using the patient-level data set split.

The choice of number and maximum depth of trees again showed only a minimal impact on performance on V2 (Figure 6a). Furthermore, all input feature sets resulted in gene selections with poor performance on the validation data (Figure 6b). For the best configuration in the final round of feature selection we chose the top 4,000 HVGs since they showed the highest mean F1-score among all sets that were not chosen at random.

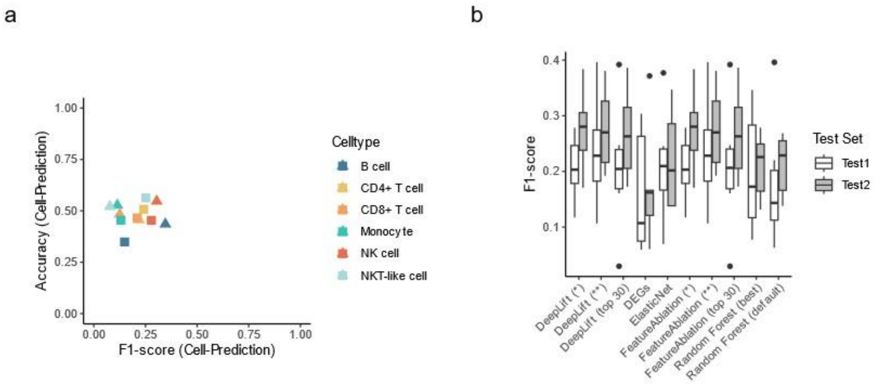

Figure 7: Result of the classification on the patient-level split. a) Accuracy and F1 score of the Random Forest method are similar on Test 1 and Test 2. b) Lower F1 score of the different feature selections when classified with a kNN. (*) and (**) as in Figure 4.

None of the applied methods were able to identify stable gene signatures predictive of Alzheimer's disease in the data. Gene selections from all machine learning approaches as well as DEGs did not generalize well (mean F1-score < 0.3) for predictions on the test sets with independent donors (Figure 7).

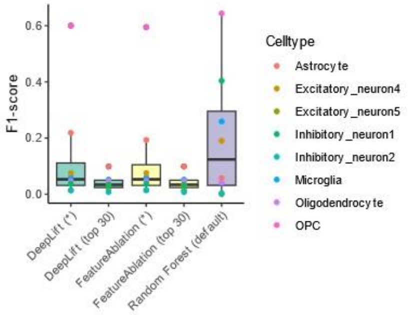

Figure 8: F1 score of the classification of the ZEBRA dataset using the features selected by the different methods.

Arguing that a stronger signal may be exhibited in brain tissue than in blood cells, we additionally evaluated our method on a data set from brains of Alzheimer's patients. For this we used the ZEBRA atlas which includes data from several Alzheimer's studies (also used for Figure 5 of the manuscript) allowing us to use an independent study for testing. The F1 scores of the classification results were below 0.2 for most methods and cell types. We were able to classify OPCs with an F1 score up to 0.64 (Figure 8). Independent of the method, the classification results were rather low.

Our results indicate that classification in the context of Alzheimer's disease is not a good application of machine learning based feature selection methods as of now due to the complexity of the problem. Furthermore, the results also highlight the importance of splitting the training/validation and test data by patient, sample or ideally even by study to enable unbiased evaluations of gene selections from scRNA-seq data.

Methods for Supplementary Note 1

Dataset splitting

The dataset was split as described in Figure 2 and Figure 5. The best input feature set and hyperparameters were determined using the models trained on the Training/Validation1 Set and evaluated on the Validation2 Set. 5-fold cross-validation was used for the feature-selection and the independent test sets were used for hyperparameter-optimization and testing.
The final model was trained using the hyperparameters that showed the highest mean F1-score across the six biggest cell-groups (CD4, CD8, NK, NKT-like, B and Monocytes), using the cells from Training/Validation1 and Validation2. The performance was evaluated using Training 1 and Training 2.
For the training and testing of the Brain data (ZEBRA dataset) [91], we used only cells from female donors. We identified and removed overlapping samples between the included studies to prevent data leakage. The Mathys 2019 study [92], the Sayed 2021 study [93] and the Morabito 2021 [94] study were used for feature selection while the Blanchard 2022 study [95] was used to evaluate the classification performance. Only cell types with at least 1,000 cells from each condition were considered.

Defining the Input Gene Sets

The top 2000, 4000, 6000 and 8000 highest variable genes were determined by Seurat's [96] FindVariableFeatures function. Differentially expressed genes were determined using limma-voom with a threshold of adj. p-value < 0.05 and abs(log2 FC)> 0.5. The 2000 randomly chosen genes were determined by randomly sampling from all genes in the data set. The feature set excluding only the lowly expressed genes was determined by removing features that were expressed in less than 1% of cells in each condition within the training set of a particular cell type. Normalized gene expression values were used as input for training of all machine learning methods.

Random Forest-based Feature Selection

The random forest-based feature selection approach was implemented in R using the Ranger package (version 0.14.1) [97] for random forest models and the Caret package (version 6.0-93) [98] for backwards feature elimination.

Using cross-validation, an initial random forest classifier is trained on each training fold and gene rankings are determined using the initial models Gini impurity feature importance scores (importance = "impurity"). The performance of the models is evaluated using the validation folds (V1) and the bottom 20% of genes with the lowest feature importance (always based on the importance determined by the initial model) are removed from the input data (backwards feature elimination). Training and validation are repeated with the new, decreased input feature set. The feature elimination, training and validation are iteratively performed until only 3 features are left. The final selection of features is then either the input feature subset resulting in the highest classification performance across all resampling folds (for ≤150 genes) or the smallest set performing within a tolerance threshold (0.5% for >150 genes; 1.0% for >500 genes) from the best feature-set. The final importance scores are averaged over the initial scores from all folds.

In the cell-level data set split, the approach was applied to the class-balanced (via downsampling of the majority class) training data with 5-fold cross-validation and accuracy as performance metric for evaluation of the different feature subsets.

For the patient-level data split, a group cross-validation approach (5-folds) based on donors was applied. To address the issue of potential class imbalances introduced by this approach, the cells from the majority class within each training fold were downsampled. We employed a weighted F1-score based on fold sizes to account for the varying sizes of the validation folds.

Logistic Regression with Elastic Net Regularization

Feature selection with logistic regression and elastic net regularization was performed using the glmnet R package (version 4.1-2) [99]. All genes passing the low expression filter described above were used as input features for the models. The parameter α controls the balance between the L1 (Lasso) and L2 (Ridge) regularization terms in the objective function of the regression model. It was set to 0.5 to equally incorporate Lasso and Ridge regularization. The parameter λ represents the overall strength of regularization for logistic regression. It directly relates to the number of features with coefficients set to zero during training of the model. For the cell-level data set split, λ was tuned over a grid of values using 10-fold cross-validation. In the patient-level data set split, similarly to the random forest approach, we used 5-fold group cross-validation based on donors for evaluation of the different λ values. Class weights equal to the proportion of each class were integrated in the models to address the issue of potential class imbalances introduced by the group cross-validation. For the gene ranking we used the model corresponding to the one-standard-error λ. This corresponds to the most regularized model whose performance falls within one standard deviation of the minimum cross-validated error. Only genes with non-zero coefficients were selected, as features with coefficients equal to zero do not contribute to the model.

Deep Learning-based Feature Selection

Deep learning important features (DeepLIFT) a gradient backpropagation-based feature selection method for neural networks and FeatureAblation, a model that determines the impact of each feature by replacing it with a baseline value, were applied using the publicly available implementation from Huang et al. [100]. This trains a multilayer perceptron (MLP) model with three fully connected layers on the class-balanced training data for binary classification of condition in each cell type and applies DeepLIFT and FeatureAblation to generate the importance scores. As input, the models use the genes passing the previously described low expression filter and the parameters were set as described in the manuscript.

Since these methods only provide a ranking of features but not a selection, we used the top 30 genes with highest importance scores as well as the number of top-ranking genes equal to the number of genes chosen by the random forest approach for comparison of gene selections.

Comparison of Feature Selections

A kNN (Caret-package, version 6.0-93) was used to perform model-independent comparison of the gene selections. For parameter tuning of k (between 5 and 23), the model was trained and evaluated using a 10-fold cross validation on the class-balanced training data (TR+V1 and V2)

with the gene selections of each method as input. The model performance was then assessed on the independent test sets (T1 and T2) using F1-scores.

References

1. Xiong, L.L., et al., *Single-cell RNA sequencing reveals B cell-related molecular biomarkers for Alzheimer's disease.* Exp Mol Med, 2021. **53**(12): p. 1888-1901.
2. Hu, G.R., et al., *Peripheral blood lymphocyte subset distribution and function in patients with Alzheimer's disease and other dementias.* Aust N Z J Med, 1995. **25**(3): p. 212-7.
3. Pirttila, T., S. Mattinen, and H. Frey, *The decrease of CD8-positive lymphocytes in Alzheimer's disease.* J Neurol Sci, 1992. **107**(2): p. 160-5.
4. Gate, D., et al., *Clonally expanded CD8 T cells patrol the cerebrospinal fluid in Alzheimer's disease.* Nature, 2020. **577**(7790): p. 399-404.
5. Shad, K.F., et al., *Peripheral markers of Alzheimer's disease: surveillance of white blood cells.* Synapse, 2013. **67**(8): p. 541-3.
6. Wang, X., et al., *Sodium oligomannate therapeutically remodels gut microbiota and suppresses gut bacterial amino acids-shaped neuroinflammation to inhibit Alzheimer's disease progression.* Cell Res, 2019. **29**(10): p. 787-803.
7. Amin, J., et al., *Peripheral immunophenotype in dementia with Lewy bodies and Alzheimer's disease: an observational clinical study.* J Neurol Neurosurg Psychiatry, 2020. **91**(11): p. 1219-1226.
8. Qian, X.H., et al., *Integrating peripheral blood and brain transcriptomics to identify immunological features associated with Alzheimer's disease in mild cognitive impairment patients.* Front Immunol, 2022. **13**: p. 986346.
9. Li, R., et al., *Abnormal B-Cell and Tfh-Cell Profiles in Patients With Parkinson Disease: A Cross-sectional Study.* Neurol Neuroimmunol Neuroinflamm, 2022. **9**(2).
10. Chen, X., et al., *Evidence for Peripheral Immune Activation in Parkinson's Disease.* Front Aging Neurosci, 2021. **13**: p. 617370.
11. Rocha, N.P., et al., *Reduced Activated T Lymphocytes (CD4+CD25+) and Plasma Levels of Cytokines in Parkinson's Disease.* Mol Neurobiol, 2018. **55**(2): p. 1488-1497.
12. Garfias, S., et al., *Peripheral blood lymphocyte phenotypes in Alzheimer and Parkinson's diseases.* Neurologia (Engl Ed), 2022. **37**(2): p. 110-121.
13. Kustrimovic, N., et al., *Parkinson's disease patients have a complex phenotypic and functional Th1 bias: cross-sectional studies of CD4+ Th1/Th2/T17 and Treg in drug-naive and drug-treated patients.* J Neuroinflammation, 2018. **15**(1): p. 205.
14. Sochocka, M., et al., *Sex Differences in Innate Immune Response of Peripheral Blood Leukocytes of Alzheimer's Disease Patients.* Arch Immunol Ther Exp (Warsz), 2022. **70**(1): p. 16.
15. Ji, W., et al., *Bioinformatics analysis of diagnostic biomarkers for Alzheimer's disease in peripheral blood based on sex differences and support vector machine algorithm.* Hereditas, 2022. **159**(1): p. 38.
16. Coales, I., et al., *Alzheimer's disease-related transcriptional sex differences in myeloid cells.* J Neuroinflammation, 2022. **19**(1): p. 247.
17. Patel, S., et al., *Donor-Specific Transcriptomic Analysis of Alzheimer's Disease-Associated Hypometabolism Highlights a Unique Donor, Ribosomal Proteins and Microglia.* eNeuro, 2020. **7**(6).
18. Felsky, D., et al., *The Caribbean-Hispanic Alzheimer's disease brain transcriptome reveals ancestry-specific disease mechanisms.* Neurobiol Dis, 2023. **176**: p. 105938.
19. Zhao, Y., et al., *Identification of Biomarkers Associated With Alzheimer's Disease by Bioinformatics Analysis.* Am J Alzheimers Dis Other Demen, 2016. **31**(2): p. 163-8.
20. Moradifard, S., et al., *Analysis of microRNA and Gene Expression Profiles in Alzheimer's Disease: A Meta-Analysis Approach.* Sci Rep, 2018. **8**(1): p. 4767.

21. Carlisle, S.M., et al., *Sex-based differences in the activation of peripheral blood monocytes in early Parkinson disease.* NPJ Parkinsons Dis, 2021. **7**(1): p. 36.
22. Sommer, A., et al., *Th17 Lymphocytes Induce Neuronal Cell Death in a Human iPSC-Based Model of Parkinson's Disease.* Cell Stem Cell, 2018. **23**(1): p. 123-131 e6.
23. Mogi, M., et al., *Caspase activities and tumor necrosis factor receptor R1 (p55) level are elevated in the substantia nigra from parkinsonian brain.* J Neural Transm (Vienna), 2000. **107**(3): p. 335-41.
24. Abdulwahid Arif, I. and H. Ahmad Khan, *Environmental toxins and Parkinson's disease: putative roles of impaired electron transport chain and oxidative stress.* Toxicol Ind Health, 2010. **26**(2): p. 121-8.
25. Lang, C., et al., *Single-Cell Sequencing of iPSC-Dopamine Neurons Reconstructs Disease Progression and Identifies HDAC4 as a Regulator of Parkinson Cell Phenotypes.* Cell Stem Cell, 2019. **24**(1): p. 93-106 e6.
26. Varghese, M., et al., *Reduced NADH coenzyme Q dehydrogenase activity in platelets of Parkinson's disease, but not Parkinson plus patients, from an Indian population.* J Neurol Sci, 2009. **279**(1-2): p. 39-42.
27. Holmes, S., et al., *Effects of Oxidative Stress and Testosterone on Pro-Inflammatory Signaling in a Female Rat Dopaminergic Neuronal Cell Line.* Endocrinology, 2016. **157**(7): p. 2824-35.
28. Maki, M., et al., *Decreased expression of hippocampal cholinergic neurostimulating peptide precursor protein mRNA in the hippocampus in Alzheimer disease.* J Neuropathol Exp Neurol, 2002. **61**(2): p. 176-85.
29. Tsugu, Y., et al., *High levels of hippocampal cholinergic neurostimulating peptide (HCNP) in the CSF of some patients with Alzheimer's disease.* Eur J Neurol, 1998. **5**(6): p. 561-569.
30. Nilsson, J., et al., *Cerebrospinal fluid biomarker panel for synaptic dysfunction in Alzheimer's disease.* Alzheimers Dement (Amst), 2021. **13**(1): p. e12179.
31. Kish, S.J., et al., *Brain glyceraldehyde-3-phosphate dehydrogenase activity in human trinucleotide repeat disorders.* Arch Neurol, 1998. **55**(10): p. 1299-304.
32. Butterfield, D.A., S.S. Hardas, and M.L. Lange, *Oxidatively modified glyceraldehyde-3-phosphate dehydrogenase (GAPDH) and Alzheimer's disease: many pathways to neurodegeneration.* J Alzheimers Dis, 2010. **20**(2): p. 369-93.
33. Tsai, C.W., et al., *An investigation of the correlation between the S-glutathionylated GAPDH levels in blood and Alzheimer's disease progression.* PLoS One, 2020. **15**(5): p. e0233289.
34. Dang, Y., et al., *FTH1- and SAT1-Induced Astrocytic Ferroptosis Is Involved in Alzheimer's Disease: Evidence from Single-Cell Transcriptomic Analysis.* Pharmaceuticals (Basel), 2022. **15**(10).
35. Sirkis, D.W., et al., *Expansion of highly interferon-responsive T cells in early-onset Alzheimer's disease.* bioRxiv, 2024.
36. Wang, P., et al., *Global Characterization of Peripheral B Cells in Parkinson's Disease by Single-Cell RNA and BCR Sequencing.* Front Immunol, 2022. **13**: p. 814239.
37. Wang, P., et al., *Single-cell transcriptome and TCR profiling reveal activated and expanded T cell populations in Parkinson's disease.* Cell Discov, 2021. **7**(1): p. 52.
38. Yan, S., et al., *Single-cell transcriptomics reveals the interaction between peripheral CD4(+) CTLs and mesencephalic endothelial cells mediated by IFNG in Parkinson's disease.* Comput Biol Med, 2023. **158**: p. 106801.
39. Xu, H. and J. Jia, *Single-Cell RNA Sequencing of Peripheral Blood Reveals Immune Cell Signatures in Alzheimer's Disease.* Front Immunol, 2021. **12**: p. 645666.
40. Lu, Y., et al., *Expression of Immune Related Genes and Possible Regulatory Mechanisms in Alzheimer's Disease.* Front Immunol, 2021. **12**: p. 768966.

41. Zhang, X., et al., *An X Chromosome Transcriptome Wide Association Study Implicates ARMCX6 in Alzheimer's Disease.* bioRxiv, 2023.
42. Koks, S., et al., *Longitudinal intronic RNA-Seq analysis of Parkinson's disease patients reveals disease-specific nascent transcription.* Exp Biol Med (Maywood), 2022. **247**(11): p. 945-957.
43. Craig, D.W., et al., *RNA sequencing of whole blood reveals early alterations in immune cells and gene expression in Parkinson's disease.* Nat Aging, 2021. **1**(8): p. 734-747.
44. Riboldi, G.M., et al., *Transcriptome deregulation of peripheral monocytes and whole blood in GBA-related Parkinson's disease.* Mol Neurodegener, 2022. **17**(1): p. 52.
45. Song, L., et al., *Cellular transcriptional alterations of peripheral blood in Alzheimer's disease.* BMC Med, 2022. **20**(1): p. 266.
46. Zhong, H., et al., *Using blood transcriptome analysis for Alzheimer's disease diagnosis and patient stratification.* Alzheimers Dement, 2024. **20**(4): p. 2469-2484.
47. Griswold, A.J., et al., *Immune and Inflammatory Pathways Implicated by Whole Blood Transcriptomic Analysis in a Diverse Ancestry Alzheimer's Disease Cohort.* J Alzheimers Dis, 2020. **76**(3): p. 1047-1060.
48. Gardner, O.K., et al., *RNA editing alterations in a multi-ethnic Alzheimer disease cohort converge on immune and endocytic molecular pathways.* Hum Mol Genet, 2019. **28**(18): p. 3053-3061.
49. Hooshmand, K., et al., *Overlap between Central and Peripheral Transcriptomes in Parkinson's Disease but Not Alzheimer's Disease.* Int J Mol Sci, 2022. **23**(9).
50. Song, L., et al., *Impaired type I interferon signaling activity implicated in the peripheral blood transcriptome of preclinical Alzheimer's disease.* EBioMedicine, 2022. **82**: p. 104175.
51. Panitch, R., et al., *Blood and brain transcriptome analysis reveals APOE genotype-mediated and immune-related pathways involved in Alzheimer disease.* Alzheimers Res Ther, 2022. **14**(1): p. 30.
52. Hemmings, S.M.J., et al., *RNA-seq analysis of gene expression profiles in posttraumatic stress disorder, Parkinson's disease and schizophrenia identifies roles for common and distinct biological pathways.* Discov Ment Health, 2022. **2**(1): p. 6.
53. Dressman, D., et al., *Genotype-phenotype correlation of T-cell subtypes reveals senescent and cytotoxic genes in Alzheimer's disease.* Hum Mol Genet, 2022. **31**(19): p. 3355-3366.
54. Infante, J., et al., *Identification of candidate genes for Parkinson's disease through blood transcriptome analysis in LRRK2-G2019S carriers, idiopathic cases, and controls.* Neurobiol Aging, 2015. **36**(2): p. 1105-9.
55. Dhanwani, R., et al., *Transcriptional analysis of peripheral memory T cells reveals Parkinson's disease-specific gene signatures.* NPJ Parkinsons Dis, 2022. **8**(1): p. 30.
56. Infante, J., et al., *Comparative blood transcriptome analysis in idiopathic and LRRK2 G2019S-associated Parkinson's disease.* Neurobiol Aging, 2016. **38**: p. 214 e1-214 e5.
57. Annesley, S.J., et al., *Dysregulated Gene Expression in Lymphoblasts from Parkinson's Disease.* Proteomes, 2022. **10**(2).
58. Garofalo, M., et al., *Alzheimer's, Parkinson's Disease and Amyotrophic Lateral Sclerosis Gene Expression Patterns Divergence Reveals Different Grade of RNA Metabolism Involvement.* Int J Mol Sci, 2020. **21**(24).
59. Henderson, A.R., et al., *DNA Methylation and Expression Profiles of Whole Blood in Parkinson's Disease.* Front Genet, 2021. **12**: p. 640266.
60. Kurvits, L., et al., *Transcriptomic profiles in Parkinson's disease.* Exp Biol Med (Maywood), 2021. **246**(5): p. 584-595.
61. Hu, Y., et al., *Exercise Reverses Dysregulation of T-Cell-Related Function in Blood Leukocytes of Patients With Parkinson's Disease.* Front Neurol, 2019. **10**: p. 1389.

62. Cardona, K., et al., *Inflammatory gene expression profiling in peripheral blood from patients with Alzheimer's disease reveals key pathways and hub genes with potential diagnostic utility: a preliminary study.* PeerJ, 2021. **9**: p. e12016.
63. Schlachetzki, J.C.M., et al., *A monocyte gene expression signature in the early clinical course of Parkinson's disease.* Sci Rep, 2018. **8**(1): p. 10757.
64. Wang, L., et al., *Transcriptome analysis reveals potential marker genes for diagnosis of Alzheimer's disease and vascular dementia.* Front Genet, 2022. **13**: p. 1038585.
65. Haage, V. and P.L. De Jager, *Neuroimmune contributions to Alzheimer's disease: a focus on human data.* Mol Psychiatry, 2022. **27**(8): p. 3164-3181.
66. Katdare, K.A., et al., *IQGAP2 regulates blood-brain barrier immune dynamics.* bioRxiv, 2024.
67. Li, H. and R. Wang, *A focus on CXCR4 in Alzheimer's disease.* Brain Circ, 2017. **3**(4): p. 199-203.
68. Bryan, K.J., et al., *Expression of CD74 is increased in neurofibrillary tangles in Alzheimer's disease.* Mol Neurodegener, 2008. **3**: p. 13.
69. Su, H., et al., *The biological function and significance of CD74 in immune diseases.* Inflamm Res, 2017. **66**(3): p. 209-216.
70. Guillot-Sestier, M.V. and T. Town, *Innate immunity in Alzheimer's disease: a complex affair.* CNS Neurol Disord Drug Targets, 2013. **12**(5): p. 593-607.
71. Corbett, B.F., et al., *DeltaFosB Regulates Gene Expression and Cognitive Dysfunction in a Mouse Model of Alzheimer's Disease.* Cell Rep, 2017. **20**(2): p. 344-355.
72. Ling, Y., et al., *The mechanism of mitochondrial metabolic gene PMAIP1 involved in Alzheimer's disease process based on bioinformatics analysis and experimental validation.* Clinics (Sao Paulo), 2024. **79**: p. 100373.
73. Liu, W., et al., *The Inflammatory Gene PYCARD of the Entorhinal Cortex as an Early Diagnostic Target for Alzheimer's Disease.* Biomedicines, 2023. **11**(1).
74. Hou, J., et al., *Antibody-mediated targeting of human microglial leukocyte Ig-like receptor B4 attenuates amyloid pathology in a mouse model.* Sci Transl Med, 2024. **16**(741): p. eadj9052.
75. Roy, E.R., et al., *Type I interferon response drives neuroinflammation and synapse loss in Alzheimer disease.* J Clin Invest, 2020. **130**(4): p. 1912-1930.
76. Shigemizu, D., et al., *Identification of potential blood biomarkers for early diagnosis of Alzheimer's disease through RNA sequencing analysis.* Alzheimers Res Ther, 2020. **12**(1): p. 87.
77. Bellenguez, C., et al., *New insights into the genetic etiology of Alzheimer's disease and related dementias.* Nat Genet, 2022. **54**(4): p. 412-436.
78. Altinoz, M.A., et al., *Involvement of hemoglobins in the pathophysiology of Alzheimer's disease.* Exp Gerontol, 2019. **126**: p. 110680.
79. Kamphuis, W., et al., *GFAP and vimentin deficiency alters gene expression in astrocytes and microglia in wild-type mice and changes the transcriptional response of reactive glia in mouse model for Alzheimer's disease.* Glia, 2015. **63**(6): p. 1036-56.
80. Ries, M., et al., *The anti-inflammatory Annexin A1 induces the clearance and degradation of the amyloid-beta peptide.* J Neuroinflammation, 2016. **13**(1): p. 234.
81. Garcia-Casas, P., et al., *Mitochondrial Ca(2+) signaling and Alzheimer's disease: Too much or too little?* Cell Calcium, 2023. **113**: p. 102757.
82. Suzuki, M., et al., *Upregulation of ribosome complexes at the blood-brain barrier in Alzheimer's disease patients.* J Cereb Blood Flow Metab, 2022. **42**(11): p. 2134-2150.
83. Gwon, Y., et al., *Amelioration of amyloid beta-FcgammaRIIb neurotoxicity and tau pathologies by targeting LYN.* FASEB J, 2019. **33**(3): p. 4300-4313.
84. Sierksma, A., et al., *Novel Alzheimer risk genes determine the microglia response to amyloid-beta but not to TAU pathology.* EMBO Mol Med, 2020. **12**(3): p. e10606.

85. Pulliam, L., et al., *Microfluidic Isolation of Neuronal-Enriched Extracellular Vesicles Shows Distinct and Common Neurological Proteins in Long COVID, HIV Infection and Alzheimer's Disease.* Int J Mol Sci, 2024. **25**(7).
86. Lu, Y., et al., *Multi-transcriptomics reveals brain cellular responses to peripheral infection in Alzheimer's disease model mice.* Cell Rep, 2023. **42**(7): p. 112785.
87. Qi, C., et al., *Alzheimer's disease alters the transcriptomic profile of natural killer cells at single-cell resolution.* Front Immunol, 2022. **13**: p. 1004885.
88. Hou, Y., et al., *Urolithin A improves Alzheimer's disease cognition and restores mitophagy and lysosomal functions.* Alzheimers Dement, 2024. **20**(6): p. 4212-4233.
89. Liu, Z., H. Li, and S. Pan, *Discovery and Validation of Key Biomarkers Based on Immune Infiltrates in Alzheimer's Disease.* Front Genet, 2021. **12**: p. 658323.
90. Brennecke, P., et al., *Accounting for technical noise in single-cell RNA-seq experiments.* Nat Methods, 2013. **10**(11): p. 1093-5.
91. Flotho, M., et al., *ZEBRA: a hierarchically integrated gene expression atlas of the murine and human brain at single-cell resolution.* Nucleic Acids Res, 2024. **52**(D1): p. D1089-D1096.
92. Mathys, H., et al., *Single-cell transcriptomic analysis of Alzheimer's disease.* Nature, 2019. **570**(7761): p. 332-337.
93. Sayed, F.A., et al., *AD-linked R47H-TREM2 mutation induces disease-enhancing microglial states via AKT hyperactivation.* Sci Transl Med, 2021. **13**(622): p. eabe3947.
94. Morabito, S., et al., *Single-nucleus chromatin accessibility and transcriptomic characterization of Alzheimer's disease.* Nat Genet, 2021. **53**(8): p. 1143-1155.
95. Blanchard, J.W., et al., *APOE4 impairs myelination via cholesterol dysregulation in oligodendrocytes.* Nature, 2022. **611**(7937): p. 769-779.
96. Hao, Y., et al., *Dictionary learning for integrative, multimodal and scalable single-cell analysis.* Nat Biotechnol, 2024. **42**(2): p. 293-304.
97. Wright, M.N. and A. Ziegler, *ranger: A Fast Implementation of Random Forests for High Dimensional Data in C plus plus and R.* Journal of Statistical Software, 2017. **77**(1): p. 1-17.
98. Kuhn, M., *Building Predictive Models in R Using the caret Package.* Journal of Statistical Software, 2008. **28**(5): p. 1-26.
99. Friedman, J., T. Hastie, and R. Tibshirani, *Regularization Paths for Generalized Linear Models via Coordinate Descent.* J Stat Softw, 2010. **33**(1): p. 1-22.
100. Huang, H., et al., *Evaluation of deep learning-based feature selection for single-cell RNA sequencing data analysis.* Genome Biol, 2023. **24**(1): p. 259.
