# Supplementary material for: A single-cell atlas to map sex-specific gene-expression changes in blood upon neurodegeneration
Source: Nat Commun. 2025 Feb 25;16:1965. doi: 10.1038/s41467-025-56833-7 (PMC11862118; doi:10.1038/s41467-025-56833-7)
Supplement: Supplementary file 2 — Description of Additional Supplementary Files [file 41467_2025_56833_MOESM2_ESM.pdf]

Description of Additional Supplementary Files:

Supplementary Data 1: Metadata
Metadata of patients and biological samples from the ADRC cohort used for the single-cell profiling.

Supplementary Data 2: DEGs of the PBMC data
Statistics for the differentially expression analysis per cell type, comparison and sex.

Supplementary Data 3: Pathway Analysis of the PBMC data
Significantly enriched or depleted pathways for each cell-type and comparison.

Supplementary Data 4: DEGs of the ZEBRA Brain data
Significantly de-regulated genes in the brain data from the ZEBRA dataset.

Supplementary Data 5: DEGs of the ROSMAP Brain data
Significantly de-regulated genes in the brain data from the ROSMAP dataset.

Supplementary Data 6: Pathway Analysis of the ZEBRA Brain data
Result of the pathway analysis for the brain cells of the ZEBRA dataset split by sex.

Supplementary Data 7: Pathway Analysis of the ROSMAP Brain data
Result of the pathway analysis for the brain cells of the ROSMAP dataset split by sex.

Supplementary Data 8: Random Forest Feature Selection
Detailed results of the Random Forest Feature Selection described in Supplementary Note 1.
