# Supplementary material for: A single-cell atlas to map sex-specific gene-expression changes in blood upon neurodegeneration
Source: Nat Commun. 2025 Feb 25;16:1965. doi: 10.1038/s41467-025-56833-7 (PMC11862118; doi:10.1038/s41467-025-56833-7)
Supplement: Supplementary file 11 — Reporting Summary [file 41467_2025_56833_MOESM11_ESM.pdf]

nature portfolio

Corresponding author(s): Andreas Keller, Tony Wyss-Coray

Last updated by author(s): Jan 13, 2025

Reporting Summary

Nature Portfolio wishes to improve the reproducibility of the work that we publish. This form provides structure for consistency and transparency in reporting. For further information on Nature Portfolio policies, see our Editorial Policies and the Editorial Policy Checklist.

Statistics

For all statistical analyses, confirm that the following items are present in the figure legend, table legend, main text, or Methods section.

n/a	Confirmed	
☐	☒	The exact sample size (n) for each experimental group/condition, given as a discrete number and unit of measurement
☐	☒	A statement on whether measurements were taken from distinct samples or whether the same sample was measured repeatedly
☐	☒	The statistical test(s) used AND whether they are one- or two-sided<br>*Only common tests should be described solely by name; describe more complex techniques in the Methods section.*
☐	☒	A description of all covariates tested
☐	☒	A description of any assumptions or corrections, such as tests of normality and adjustment for multiple comparisons
☐	☒	A full description of the statistical parameters including central tendency (e.g. means) or other basic estimates (e.g. regression coefficient) AND variation (e.g. standard deviation) or associated estimates of uncertainty (e.g. confidence intervals)
☐	☒	For null hypothesis testing, the test statistic (e.g. F, t, r) with confidence intervals, effect sizes, degrees of freedom and P value noted<br>*Give P values as exact values whenever suitable.*
☒	☐	For Bayesian analysis, information on the choice of priors and Markov chain Monte Carlo settings
☒	☐	For hierarchical and complex designs, identification of the appropriate level for tests and full reporting of outcomes
☐	☒	Estimates of effect sizes (e.g. Cohen's d, Pearson's r), indicating how they were calculated

Our web collection on statistics for biologists contains articles on many of the points above.

Software and code

Policy information about availability of computer code

Data collection	No software was used for data collection
Data analysis	Code written to analyze the data is available from GitHub.

For manuscripts utilizing custom algorithms or software that are central to the research but not yet described in published literature, software must be made available to editors and reviewers. We strongly encourage code deposition in a community repository (e.g. GitHub). See the Nature Portfolio guidelines for submitting code & software for further information.

Data

Policy information about availability of data

All manuscripts must include a data availability statement. This statement should provide the following information, where applicable:

- Accession codes, unique identifiers, or web links for publicly available datasets
- A description of any restrictions on data availability
- For clinical datasets or third party data, please ensure that the statement adheres to our policy

Raw sequencing data is freely available from the sequence read archive (SRA) using accession ID SRP312418. An interactive web server to explore the scRNA-seq count data is freely avaliable at https://www.ccb.uni-saarland.de/adrcsc.

Research involving human participants, their data, or biological material

Policy information about studies with human participants or human data. See also policy information about sex, gender (identity/presentation), and sexual orientation and race, ethnicity and racism.

Reporting on sex and gender	The study included the self-reported sex of the pariticipants.
Reporting on race, ethnicity, or other socially relevant groupings	The dataset includes self-reported values by the participants for the race based on the cathegories American Indian/Alaska Native, Asian, Black or African American, Native Hawaiian or other Pacific Islander and White and the options for more than one race and unknown/not reported.
Population characteristics	The dataset includes patients with Alzhimer's disease, Parkinson's disease, Mild Cognitive Impairment and Healthy patients. The age ranges from 26-96 years.
Recruitment	The patients were enrolled by the Stanford Alzhiemer's Disease Research Center (ADRC)
Ethics oversight	The study was approved by the Stanford University IRB

Note that full information on the approval of the study protocol must also be provided in the manuscript.

Field-specific reporting

Please select the one below that is the best fit for your research. If you are not sure, read the appropriate sections before making your selection.

[x] Life sciences [ ] Behavioural & social sciences [ ] Ecological, evolutionary & environmental sciences

For a reference copy of the document with all sections, see nature.com/documents/nr-reporting-summary-flat.pdf

Life sciences study design

All studies must disclose on these points even when the disclosure is negative.

Sample size	The final dataset (after quality control) included 290 samples form 257 individuals.
Data exclusions	73 samples were removed from the dataset due to quality concerns (based on the quality-control described in the paper).
Replication	We compared our findings with previously published papers and were able to confirm some, but not all of our findings. We clearly state this in the manuscript.
Randomization	This does not apply to our study, as the patients were assigned the groups based on their diagnosis.
Blinding	Blinding is not relevant to our study as we compare different diagnosis.

Reporting for specific materials, systems and methods

We require information from authors about some types of materials, experimental systems and methods used in many studies. Here, indicate whether each material, system or method listed is relevant to your study. If you are not sure if a list item applies to your research, read the appropriate section before selecting a response.

Materials & experimental systems

n/a	Involved in the study	
[ ]	[x]	Antibodies
[x]	[ ]	Eukaryotic cell lines
[x]	[ ]	Palaeontology and archaeology
[x]	[ ]	Animals and other organisms
[x]	[ ]	Clinical data
[x]	[ ]	Dual use research of concern
[x]	[ ]	Plants

Methods

n/a	Involved in the study	
[x]	[ ]	ChIP-seq
[x]	[ ]	Flow cytometry
[x]	[ ]	MRI-based neuroimaging

Antibodies

Antibodies used	CSF markers were measured using antibodies for NFL, UCHL1, Tau and GFAP (Quanterix4plex), Ab40, Ab42 and Tau (Quanterix3plex) and pTau181 (Quanterix).

Validation	Validation was performed by the manufacturer.

Plants

Seed stocks	*Report on the source of all seed stocks or other plant material used. If applicable, state the seed stock centre and catalogue number. If plant specimens were collected from the field, describe the collection location, date and sampling procedures.*
Novel plant genotypes	*Describe the methods by which all novel plant genotypes were produced. This includes those generated by transgenic approaches, gene editing, chemical/radiation-based mutagenesis and hybridization. For transgenic lines, describe the transformation method, the number of independent lines analyzed and the generation upon which experiments were performed. For gene-edited lines, describe the editor used, the endogenous sequence targeted for editing, the targeting guide RNA sequence (if applicable) and how the editor was applied.*
Authentication	*Describe any authentication procedures for each seed stock used or novel genotype generated. Describe any experiments used to assess the effect of a mutation and, where applicable, how potential secondary effects (e.g. second site T-DNA insertions, mosiacism, off-target gene editing) were examined.*
